# Measuring the Cohesive Law in Mode I Loading of *Eucalyptus globulus*

**DOI:** 10.3390/ma12010023

**Published:** 2018-12-21

**Authors:** Almudena Majano-Majano, Antonio José Lara-Bocanegra, José Xavier, José Morais

**Affiliations:** 1 Department of Building Structures and Physics, ETS of Architecture, Universidad Politécnica de Madrid, Avda Juan de Herrera 4, 28040 Madrid, Spain; antoniojose.lara@upm.es; 2 Department of Mechanical and Industrial Engineering, UNIDEMI, Faculty of Sciences and Technology, New University of Lisbon, 2928-516 Caparica, Portugal; jmc.xavier@fct.unl.pt; 3 School of Science and Technology, CITAB, University of Trás-os-Montes e Alto Douro, 5001-801 Vila Real, Portugal; jmorais@utad.pt

**Keywords:** *Eucalyptus globulus*, cohesive law, double cantilever beam, compliance-based beam method, mode I, digital image correlation

## Abstract

Assessing wood fracture behavior is essential in the design of structural timber elements and connections. This is particularly the case for connections with the possibility of brittle splitting failure. The numerical cohesive zone models that are used to simulate the fracture behavior of wood make it necessary to assume a cohesive law of the material that relates cohesive tractions and crack opening displacements ahead of the crack tip. This work addresses the determination of the fracture cohesive laws of *Eucalyptus globulus*, a hardwood species with great potential in timber engineering. This study centres on Mode I fracture loading for RL and TL crack propagation systems using Double Cantilever Beam tests. The Compliance-Based Beam Method is applied as the data reduction scheme in order to obtain the strain energy release rate from the load-displacement curves. The cohesive laws are determined by differentiating the relationship between strain energy release rate and crack tip opening displacement. The latter is measured by the digital image correlation technique. High strain energy release rates were obtained for this species, with no big differences between crack propagation systems. The difference between the crack systems is somewhat more pronounced in terms of maximum stress that determines the respective cohesive laws.

## 1. Introduction

Hardwood species are increasingly used for structural purposes, and this is shown, for instance, by in the development of new products with great impact in the European market. In particular, *Eucalyptus globulus* Labill is seen as a hardwood species with major potential in timber engineering, because of its high mechanical performance and durability, its aesthetic qualities, and the large stock of eucalyptus resources. This situation requires continuous research in different fields, where improvement of the drying process and the development of laminated products are two of the main ongoing research focuses [1,2,3,4].

Fracture characterization of wood is of essential importance, especially in the design of timber elements and connections in engineering structures [5,6]. Connections are most particularly some of the most critical parts, as they may lead to a dangerous situation in cases of brittle splitting failure produced by tension perpendicular to the grain. 

The current European design formulation for the prediction of the splitting failure of dowel connections loaded perpendicularly to the grain is only valid for softwood, and it is based on an energetic approach in the framework of Fracture Mechanics [7]. This formulation was originally calibrated using strain energy release rate values (*G*_I_) that were obtained from experimental research reported in the literature [8]. Although proposals of other different analytical models considering *G*_I_ as input material parameter have been compiled in the literature, [9] there is, at present, no general agreement between the results. This empirical research technique is time consuming and not-always cost-efficient. It may be more efficient to achieve these objectives through numerical simulations, which show advantages in terms of effectiveness with regard to time, cost, the exactitude of results, and the possibility of conducting parametric studies, e.g., [10,11].

Regarding numerical fracture models in wood, it must be noted that fracture behavior can be affected by nonlinear phenomena such as crack-bridging and micro-cracking along a fracture process zone (FPZ) ahead of the crack tip [12]. Accordingly, an appropriate fracture characterization can be formulated by using numerical cohesive zone models (CZM) based on nonlinear fracture mechanics in order to simulate the development of significant FPZ. In this way, the whole crack growth can be properly reproduced in a way that is closer to the actual behavior of the structure. The CZM were originally formulated for elastic-plastic fracture in metals [13,14]. Crack growth and damage phenomena in wood were firstly described in [15]. According to this and more specifically in Mode I tests, there is a linear and elastic relationship between load and displacements until the load approaches a critical value, where the first damage phenomena begin to appear in the specimen. When the maximum load is reached, the FPZ starts to develop and all of the additional displacements take place there, while the material outside the zone is elastically unloaded. In CZM, the degrading mechanisms in the FPZ are assumed to keep to a discrete line (or plane) represented by a cohesive law which defines material softening behavior and considers fracture energy evolution. This cohesive law relates the cohesive tractions and the crack tip opening displacements produced at the FPZ. Although different studies discuss the effect of cohesive stiffness and strength parameters, e.g., [16,17], the most common cohesive laws that are implemented in finite element codes are simplified into linear, bilinear, and exponential relationships. Suitable identification becomes relevant and remains an open problem, since there is no well-established methodology that makes this goal possible [18]. 

One group of methods applied in the literature for this purpose are the inverse methods [19,20] whereby cohesive laws are obtained recursively through global load-displacement curves. The difference between the numerical and experimental curves is minimized by an optimization procedure that has the drawback that a cohesive law shape must be assumed a priori as this can significantly affect the fracture results [21]. Convergence to the minimum is not always guaranteed and sometimes demands sophisticated and time-consuming optimization algorithms.

Alternatively, a direct method has been proposed with the same aim but which is instead based on the relationship between strain energy release rate and crack tip opening displacements [22], which are determined independently by local measurements. This approach has the advantage of not requiring a priori cohesive law shape assumption.

In this work, the cohesive law in mode I of *Eucalyptus globulus* was directly identified using Double Cantilever Beam (DCB) tests, and it is the first such study carried out on this species. The strain energy release rate (*G*_I_) was explicitly derived from the load-displacement curves that were obtained in each test by applying the Compliance-Based Beam Method (CBBM). This method has the advantage of not requiring measurements of crack propagation during the test, which would be too difficult in practice given the material heterogeneity. An equivalent crack length (*a*_eq_) was considered instead. The cohesive law, defined as the relationship between cohesive traction tension (*σ*_I_) and crack tip opening displacement (*w*_I_), was determined by differentiating the *G*_I_-*w*_I_ relationship and applying least-squares regression analysis. The *w*_I_-parameter was measured by digital image correlation (DIC) technique. This fracture behavior was studied for two crack propagation systems, RL and TL, where the first letter indicates the direction normal to the crack plane and the second letter refers to the crack propagation direction (Longitudinal, Radial, and Tangential).

## 2. Materials and Methods

### 2.1. Raw Material

*Eucalyptus globulus* Labill from Galicia, Northwest Spain, was used in this research. The boards were kiln-dried prior to sample preparation. It is worth noting that the boards were approximately knot-free (knot diameter less than 1/20 times board width), which is a characteristic feature of this species. Each board is identified with a reference number shown in Table 1. This table also includes the boards´ densities (ρ) determined for a reference moisture content of 12%, and the corresponding static longitudinal modulus of elasticity in the grain direction (*E*_L_) resulting from edgewise bending tests under four-point loading according to EN 408:2011 [23]. 

The orthotropic average values for the radial modulus of elasticity *E*_R_ = 1820 MPa, the tangential modulus of elasticity *E*_T_ = 821 MPa, the shear modulus of elasticity in the LR plane *G*_LR_ = 1926 MPa, and the shear modulus of elasticity in the LT plane *G*_LT_ = 969 MPa, are taken from [24] using Galician *Eucalyptus globulus* with a similar density to the boards used in this study. These parameters were obtained by compression tests coupled with a stereovision system (DIC 3D). DCB specimens were prepared from these boards according to the specifications shown in Section 2.3. 

### 2.2. Compliance-Based Beam Method (CBBM)

The procedure applied to determine the cohesive law corresponds to a direct method that requires establishing the relationship between the strain energy release rate in mode I loading (*G*_I_), the crack tip opening displacement (*w*_I_), and the traction tension (*σ*_I_), according to Equation (1).

(1) $$G_{I}=\int_{0}^{w_{I}}\sigma_{I}\left({\bar{w}}_{I}\right)d{\bar{w}}_{I}$$

The cohesive law in mode I, defined as $\sigma_{I}=f(w_{I})$, can be then determined by differentiating Equation (1), as follows:

(2) $$\sigma_{I}\left(w_{I}\right)=\frac{\partial G_{I}}{\partial w_{I}}$$

This requires the accurate measurement of *G*_I_ evolution as a function of *w*_I_ in the course of an experimental fracture test (in this case a DCB test, see details in Section 2.3). The classical data reduction schemes used for this purpose are based on beam theory or compliance calibration and require crack length (*a*) measuring during testing [25]. However, the fracture process zone (FPZ) ahead of the crack tip in wood involves toughening mechanisms, such as microcracking, crack-branching, or fiber-bridging, hindering the identification of the crack tip and therefore also the *a*-measurement. To overcome this problem, the Compliance Based Beam method (CBBM) [20,26] is shown to be a suitable alternative. It is based on Timoshenko beam theory and it introduces the concept of an equivalent crack length (*a*_eq_), accounting for the FPZ effect given by *a*_eq_ = *a* + Δ + Δ*a*_FPZ_. Accordingly, compliance for a DCB specimen during crack propagation can be written as

(3) $$C=\frac{8a_{\mathrm{eq}}{}^{3}}{E_{f}Bh^{3}}+\frac{12a_{\mathrm{eq}}}{5BhG_{\mathrm{LR}}}$$

where *G*_LR_ is the shear modulus in the LR plane; *B* and *h* the specimen dimensions; and, *E_f_* the corrected flexural modulus (instead of *E*_L_) to take into account the cross-section rotation effects at the crack tip during testing and local stress concentrations. *E_f_* can be estimated from Equation (4) when considering the initial compliance (*C*_0_) and a corrected initial crack length (*a*_0_ + Δ)

(4) $$E_{f}={\left(C_{0}-\frac{12\left(a_{0}+\Delta \right)}{5BhG_{\mathrm{LR}}}\right)}^{-1}\frac{8\left(a_{0}+\Delta \right){}^{3}}{Bh^{3}}$$

where Δ represents the Williams correction term given by [27] in the form:

(5) $$\Delta =h\sqrt{\frac{E_{f}}{11G_{\mathrm{LR}}}\left[3-2{\left(\frac{\Gamma }{1+\Gamma }\right)}^{2}\right]}$$

(6) $$\Gamma =1.18\frac{\sqrt{E_{f}E_{R}}}{G_{\mathrm{LR}}}$$

An iterative process can be used to solve Equations (4)–(6) until a converged value of *E_f_* is reached. It must be noted that *E*_T_ and *G*_LT_ values should be used instead of *E*_R_ and *G*_LR_ in Equations (3)–(7) when the TL crack propagation system is considered.

The equivalent crack length, *a*_eq_, that meets the specimen compliance recorded during propagation is evaluated from a polynomial function solved with Matlab^®^ (Mathworks, Madrid, Spain), according to [20].

Let us consider the Irwin-Kies equation [28]

(7) $$G_{I}=\frac{P^{2}}{2B}\frac{dC}{da}$$

the strain energy release rate in mode I (*G*_I_) is obtained by combining Equations (3) and (7). It represents the resistance curve (*R*-curve) of the material to the crack growth.

(8) $$G_{I}=\frac{6P_{}^{2}}{B^{2}h}\left(\frac{2a_{\mathrm{eq}}^{2}}{E_{f}h^{2}}+\frac{1}{5G_{\mathrm{LR}}}\right)$$

The CBBM method has the definitive advantage of only requiring the experimental load–displacement (*P*–*δ*) curve to derive the evolution of *G*_I_ without crack length monitoring, making it less sensitive to experimental errors. The *G*_I_ is then correlated with crack tip opening displacement in mode I (*w*_I_), measured by the digital image correlation (DIC) technique during the test (see details in Section 2.3) and its derivative yields in the cohesive law expressed in Equation (2). It is therefore important to accurately evaluate the $G_{I}=f(w_{I})$ relationship. This was performed in two ways for further comparison and discussion: (a) a smoothing spline using Matlab^®^ was adjusted to the experimental curve in order to soften the noise before differentiation; (b) the *G*_I_-*w*_I_ data were fitted, in the least-square sense, by a continuous approximation function (logistic function), as follows,

(9) $$G_{I}=\frac{A_{1}-A_{2}}{1+{\left(w_{I}/w_{I,0}\right)}^{p}}+A_{2}$$

where *A*_1_, *A*_2_, *p*, and *w*_I,0_ are constants determined by regression analysis. Although this function has no particular physical meaning, it is simply a tool for the analytical differentiation that is required to obtain the cohesive law. The *A*_2_ parameter must provide an estimation of the critical strain release, as

(10) $$A_{2}=\underset{w_{I}\to \infty }{\lim}G_{I}=G_{Ic}$$

The direct approach presented in this data reduction scheme can be potentially extended to other fracture modes, including mode II by means of the end notched flexure (ENF) test [29,30].

### 2.3. Double Cantilever Beam (DCB) Test Coupled with Digital Image Correlation

**Figure 1:**
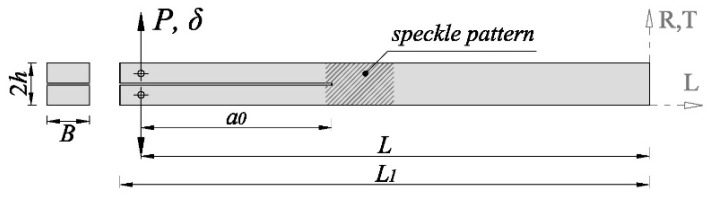
Double Cantilever Beam (DCB) specimen geometry.

Thirteen DCB specimens that were oriented along the RL crack propagation system and fourteen oriented along the TL system were prepared for fracture tests. The first letter indicates the loading direction (Radial and Tangential, respectively) and the second letter refers to the crack propagation direction (Longitudinal). The DCB specimens consist of a rectangular beam with *L*_1_ × 2*h* × *B* mm (250 mm × 20 mm × 20 mm) nominal dimension, as schematically shown in Figure 1. A mid-height pre-cracked surface of 100 mm in length and 1 mm thickness was initially performed. This initial notch was then lengthened a few millimeters with a band saw in order to guarantee a sharp initial crack. The actual *a*_0_ value for each specimen was measured after testing. A symmetrical pair of 3 mm diameter holes were drilled at 10 mm from the specimen end, where the load (*P*) perpendicular to the pre-cracked surface was applied. The applied load was transferred to the specimen by means of two 3 mm diameter steel pins that were inserted into the holes.

Prior to testing, the specimens were conditioned at 20 °C and 65% relative humidity until equilibrium moisture content was reached. The mean value of moisture content was approximately 11%.

The fracture tests were carried out using an INSTRON 1125 universal testing machine (Instron, Barcelona, Spain) with a load cell having a maximum capacity of 5 kN and 50 N/V gain. Specimens were loaded under 3 mm/min displacement control.

**Figure 2:**
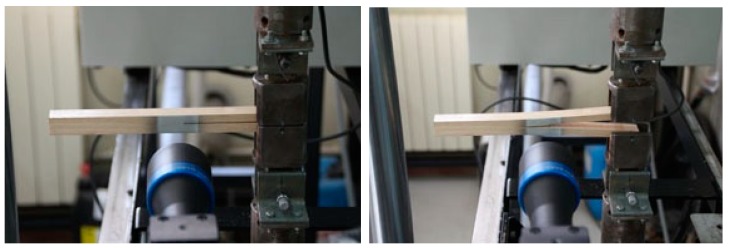
DCB test set-up coupled with digital image correlation (DIC).

Crack mouth opening displacement was recorded using the optical system ARAMIS DIC-2D (GOM mbH, Braunschweig, Germany) [31,32] (Figure 2). This is a non-contact system that applies the principles of digital image correlation (DIC). This technique shows clear advantages in comparison with traditional measurement methods since it makes it possible to measure the deformation field of a whole specimen area, providing more robust results. It is composed of an eight-bit charge-coupled device (CCD) camera with a telecentric lens that was mounted on a translation bar for fine aligning of the optical axis with regard to the planar specimen surface. The specimens were illuminated by two cold light sources incorporated in the measuring device. A speckled pattern with black ink on a white matte surface is applied to the specimen by an airbrush IWATA, model CM-B (Anesta Iwata Iberica SL, Barcelona, Spain), so that proper granulometry contrast and isotropy at the magnification scale is ensured. The region of interest is focused on the area just in front of the crack tip, where the crack starts to propagate. The different components of the optical system and the measuring parameters selected for this work are compiled in Table 2. Complete *P*-*δ* curves were obtained in all tests with an acquisition rate of 5 Hz, while the acquisition of images from DIC was made with 1 Hz frequency.

The crack tip opening displacement in mode I (*w*_I_) was obtained by post-processing the displacements monitored by DIC. The initial crack length was firstly identified in the undeformed image. The relative displacement between a pair of subsets selected close to the crack tip is evaluated afterwards. The value of *w*_I_ is calculated as the Eucledian norm, as shown in Equation (9) [33,34].

(11) $$w_{I}=\left\|w_{I}^{+}-w_{I}^{-}\right\|$$

where $w_{I}^{+}$ and $w_{I}^{-}$ are the displacement components in the direction perpendicular to the crack propagation associated to the upper and the lower cracked surface, respectively. This approach has the limitation of defining the cohesive law based on surface measurements, which may not be fully representative of the crack front over the volume of the FPZ. Current research interests have been expanded to the experimental observation of the volumetric crack front ahead of the crack tip by X-ray computed tomography [35,36] and exploring paths, which includes digital volume correlation, for the quantitative extraction of relevant mechanical [37,38] and fracture parameters [39].

## 3. Results and Discussion

### 3.1. Resistance Curve from CBBM

**Figure 3:**
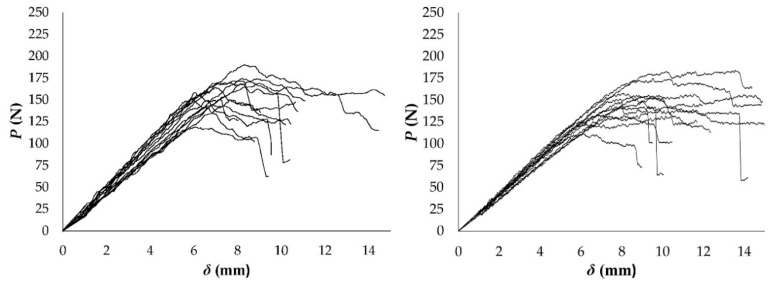
*P*-*δ* curves from DCB test in RL (**left**) and TL (**right**) propagation systems.

The load-displacement curves obtained from the DCB specimens for RL ant TL crack propagation systems are shown in Figure 3.

**Figure 4:**
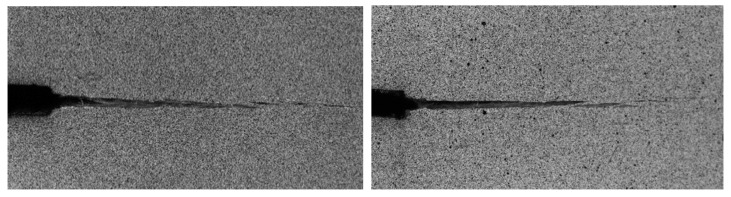
Macroscopic images of crack propagation: “DCB 176-3 RL” (**left**); “DCB 192-2 TL” (**right**).

Both groups of *P*-*δ* curves show quite consistent results with variation typical of wood. The non-linear behaviour that was observed before the curves peak reveals that a non-negligible FPZ develops ahead of the crack tip. This phenomenon is characteristic of quasi-brittle materials, like wood and results in micro-cracking and fiber bridging, as confirmed by the macroscopic image in Figure 4. These observations are in agreement with other authors’ research in wood [19,25]. This fact supports the difficulties in measuring the crack length during testing using conventional techniques and the convenience of applying the alternative CBBM method, when considering an equivalent crack length.

The maximum loads that were attained in the tests are shown in Table 3 and Table 4 for RL and TL crack propagations systems, respectively. The mean value from TL specimens is slightly lower than the one obtained from RL, but all of the values vary within a similar range. This means that the high proportion of radially oriented rays acting as reinforcement in this direction typical of hardwoods, like beech and ash [40], is not shown so pronouncedly in eucalyptus. However, microstructural constrictions may act in both directions. In most specimens, the decrease in load did not occur suddenly after reaching the peak load, as it arose gradually after a considerable increase in displacements, displaying an uneven behavior, due, once again, to the complex structure of wood [26,40]. 

**Figure 5:**
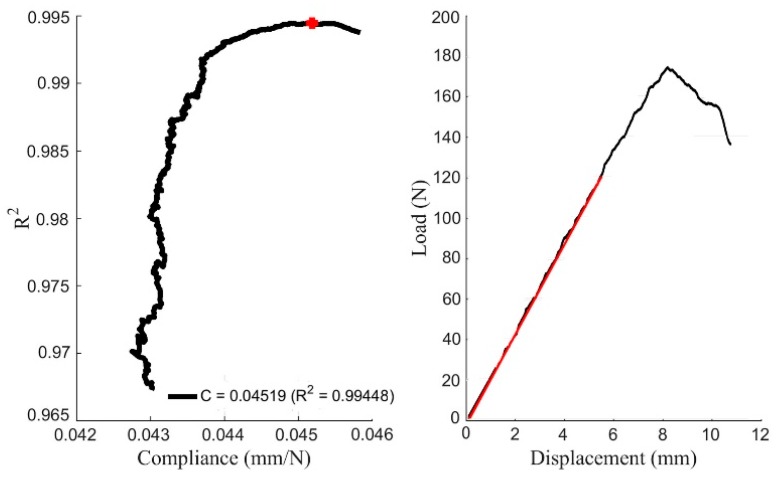
Representative curves for initial compliance determination.

The initial compliance *C*_0_ is calculated using Matlab^®^, as this is the result that provides the maximum *R*^2^ in every *P*-*δ* curve. A representative example of a DCB specimen is shown in Figure 5. The *C*_0_ values resulting from all the tests are also included in Table 3 and Table 4 for both crack propagation systems. There are minor quantitative differences between both orientations, while scatter is within the expected range for wood.

**Figure 6:**
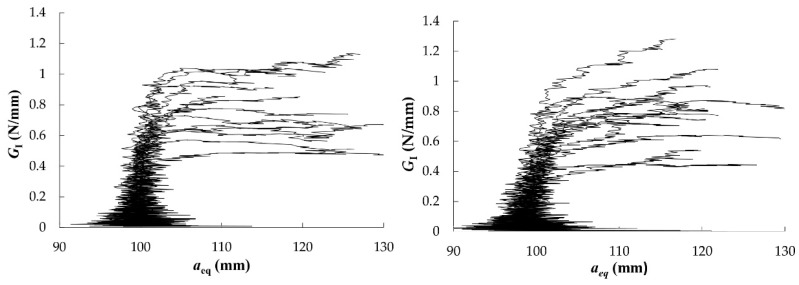
*R*-curves from DCB test in RL (**left**) and TL (**right**) propagation systems.

The *R*-curves that were obtained from DCB tests in RL and TL crack propagation systems were evaluated by applying the CBBM and they are shown in Figure 6. From them, following an initial rising domain characterized by the development of the FPZ (corresponding to the non-linearity beginning in *P*-*δ* curve), resistance to crack growth tends to a horizontal asymptote, despite the noise in the measurements, which defines the value of critical strain energy release rate (*G*_Ic_) and it represents the material´s toughness to crack-growth.

In this research, most of the specimens had plateaus for a given crack extent, which means that the FPZ has been completely developed. Therefore, the fracture energy could be determined as a mean value over the horizontal domain of the curves. In cases where the *R*-curve did not show a clear plateau, the strain energy release rate corresponding to the maximum load (*G*_I,Pmax_) could be assumed as critical strain energy release rate. Both values are reported in Table 3 and Table 4, together with the maximum load reached in the tests, the corrected flexural modulus of elasticity from every specimen and the initial compliance. The last three values are input parameters in the CBBM formulation detailed in Section 2.2. The wide dispersion of *R*-curves may be due to local variability of wood microstructure at the crack tip among the specimens, e.g., earlywood and latewood [26].

The mean *G*_Ic_ values that were obtained from both crack propagation systems were found to be the same: 0.77 N/mm. This value is considerably higher than that for other species. In particular, DCB specimens of *Pinus pinaster* gave a mean *G*_Ic_ value of 0.31 N/mm when applying the same data reduction method in [25]. *Eucalyptus globulus* also displays higher fracture energy than other hardwood species. For instance, average *G*_f_ values of 0.48 and 0.40 N/mm were obtained for beech and ash, respectively, in previous work by the author [40].

As the differences between *G*_Ic_ and *G*_I,Pmax_ were minimal (see Table 3 and Table 4), the latter can be taken as a practical measure of mode I critical strain energy release rate.

### 3.2. Cohesive Law

**Figure 7:**
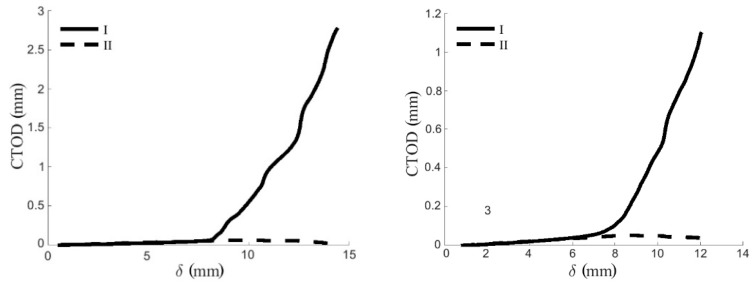
Normal and transverse crack tip opening displacements (CTOD) measured by DIC from a representative DCB test in RL (**left**) and TL (**right**) crack propagation systems.

*G*_I_ evolution was correlated with the crack tip opening displacement (CTOD) values that were provided by DIC during testing in order to obtain the cohesive law in mode I. Normal and transverse CTOD with respect to the crack plane were determined. Representative normal and transverse CTOD-*δ* curves are shown in Figure 7. As can be seen, CTOD in mode II (*w*_II_) was found to be negligible in DCB mode I tests.

**Figure 8:**
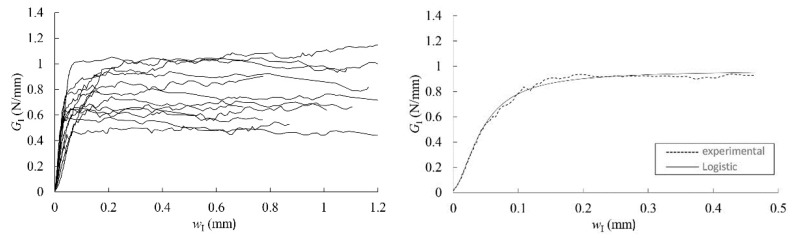
Characteristic *G*_I_-*w*_I_ curves in RL (**left**); experimental *G*_I_-*w*_I_ curve of “189-2-RL” specimen and least-square regression with the logistic function (**right**).

**Figure 9:**
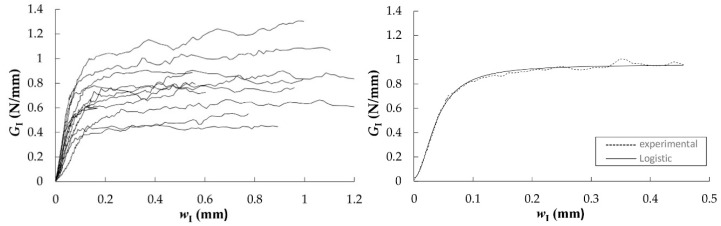
Characteristic *G*_I_-*w*_I_ curves in TL (**left**); experimental *G*_I_-*w*_I_ curve of “140-1-TL” specimen and least-square regression with the logistic function (**right**).

Characteristic *G*_I_-*w*_I_ curves were then obtained, as shown in Figure 8 and Figure 9. 

**Figure 10:**
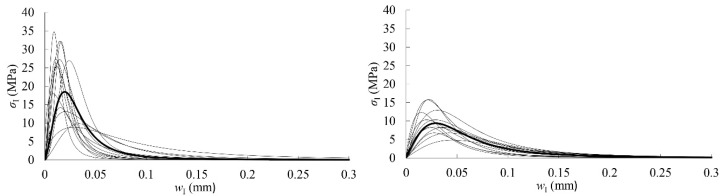
Cohesive laws in RL (**left**) and TL (**right**). Mean cohesive law is highlighted in bold.

The cohesive law for each specimen was finally determined by fitting a logistic function to the experimental data, as shown in Figure 10. The parameters corresponding to the logistic function expressed in Equation (9) (*A*_1_, *A*_2_, *p*, and *w*_I0_), the area circumscribed by the cohesive laws (*G*_law,I_), the maximum stress (*σ*_I,u_), and the relative displacements (*w*_Iu_ and *w*_Ic_) are all included in Table 5 and Table 6 for RL and TL crack propagation systems, respectively. 

In these tables, the mean value of *A*_2_ depicts an estimation of the critical strain energy release rate, *G*_Ic_, and it acquires the values of 0.78 and 0.76 N/mm in the RL and TL crack propagation systems, respectively. These values are quite close to the mean ones obtained from the horizontal asymptote of the *R*-curves, that is 0.77 N/mm in both propagation systems (see Table 3 and Table 4). 

The mean parameters of Table 5 and Table 6 are used to build the mean experimental cohesive law in mode I that can be considered for *Eucalyptus globulus* in RL and TL crack systems. It is highlighted by a bold curve in Figure 10. As can be seen, the difference between both propagation systems is more pronounced in terms of cohesive law than it is for the other previously derived fracture parameters. Mean maximum stress in RL is approximately 50% higher than in the TL system. In general, the maximum stresses that were displayed by the RL specimens are reached with lower crack tip opening displacements than in TL.

The mean cohesive laws in mode I obtained for eucalyptus could be implemented in numerical cohesive zone models to simulate the development of the FPZ and crack growth, and thereby analyze the actual fracture behavior of a timber structure.

## 4. Conclusions

The cohesive laws in mode I of *Eucalyptus globulus* for RL and TL crack propagation systems were determined by means of DCB fracture tests. The CBBM data reduction method was applied to derive the strain energy release rate (*G*_I_) from the load-displacement curves when considering an equivalent crack length (*a*_eq_) instead of the actual one, which would be difficult to measure. The *G*_I_ was correlated with the crack tip opening displacements measured by digital image correlation technique to obtain the cohesive laws.

The same *G*_Ic_ value of 0.77 N/mm was obtained for RL and TL crack propagation systems from the horizontal asymptote of the *R*-curves. The estimation of *G*_Ic_ from the logistic function parameters that was used to attain the cohesive law was also within the same range (0.78 and 0.76 N/mm in RL and TL, respectively). 

The behavioral difference between the two crack propagation systems was more significantly displayed in the cohesive laws. In this sense, RL laws showed higher values of mean maximum cohesive stress at lower crack tip opening displacements in comparison with the TL system. The cohesive laws definition in both crack propagation systems makes it possible to implement them in numerical cohesive zone models to accurately simulate crack growth along the FPZ and quantify the actual fracture behavior of the timber structure in question, especially in elements and connections involving the possibility of brittle splitting failures.

The excellent mechanical properties added to the high fracture toughness shown in this study underline that *Eucalyptus globulus* L. is a hardwood species of great interest for structural design.

## Figures and Tables

**Table 1:** Density and elastic modulus of elasticity of *Eucalyptus globulus* boards.

Board Ref	ρ (kg/m^3^)	*E*_L_ (MPa)
140	781	19,863
144	765	19,234
161	867	19,658
176	779	19,359
189	748	19,114
192	815	20,612
mean	793	19,640
SD	43	551
CoV (%)	5.4	2.8

**Table 2:** Components of the optical system and DIC measuring parameters.

CCD Camera	Settings
Model	Baumer Optronic FWX20 (8 bits, 1624 × 1236 pixels, 4.4 μm/pixel)
Shutter time	0.7 ms
Acquisition frequency	1 Hz
Lens	
Model	Opto Engineering Telecentric lens TC 23 36
Magnification	0.243 ± 3%
Field of view (1/1.8″)	29.3 × 22.1 mm^2^
Working distance	103.5 ± 3 mm
Working F-number	*f*/8
Field depth	11 mm
Conversion factor	0.018 mm/pixel
Lighting	Raylux 25 white-light LED
DIC measurements	
Subset size	15 × 15 pixel^2^ (0.270 × 0.270 mm^2^)
Subset step	13 × 13 pixel^2^ (0.234 × 0.234 mm^2^)
Resolution	1–2 × 10^−2^ pixel (0.18 × 0.36 μm)

**Table 3:** Fracture energy obtained from DCB specimens oriented in RL by means of Compliance-Based Beam Method (CBBM).

Specimen Ref	*E_f_* (MPa)	*P*_max_ (N)	*C*_0_ (mm/N)	*G*_I,Pmax_ (N/mm)	*G*_Ic_ (N/mm)
140-1-RL	14,250	191.70	0.042	1.07	1.01
144-1-RL	15,203	173.00	0.040	0.81	0.84
144-2-RL	13,254	123.08	0.048	0.52	0.48
161-1-RL	12,266	172.35	0.047	0.97	0.95
161-3-RL	14,593	176.29	0.039	0.85	0.85
176-1-RL	14,557	151.70	0.042	0.65	0.61
176-2-RL	12,335	156.59	0.049	0.82	0.76
176-3-RL	11,577	183.95	0.048	1.10	1.02
189-1-RL	15,087	168.16	0.039	0.75	0.70
189-2-RL	12,293	177.06	0.045	0.95	0.92
192-1-RL	16,707	162.01	0.038	0.65	0.63
192-2-RL	14,399	147.76	0.044	0.70	0.65
192-3-RL	16,103	153.99	0.038	0.56	0.57
Mean	14,048	164.43	0.043	0.80	0.77
SD	1590	18.03	0.004	0.19	0.18
CoV (%)	11	11	10	23	23

**Table 4:** Fracture energy obtained from DCB specimens oriented in TL by means of CBBM.

Specimen Ref	*E_f_* (MPa)	*P*_max_ (N)	*C*_0_ (mm/N)	*G*_I,Pmax_ (N/mm)	*G*_Ic_ (N/mm)
140-1-TL	14,491	182.31	0.046	1.03	0.96
140-2-TL	11,295	157.74	0.055	0.94	0.89
144-1-TL	14,080	154.80	0.049	0.75	0.72
161-1-TL	12,742	188.28	0.049	1.28	1.09
176-1-TL	12,466	146.01	0.054	0.84	0.82
176-2-TL	13,516	160.37	0.049	0.93	0.84
176-3-TL	12,103	147.33	0.053	0.81	0.78
189-1-TL	12,482	162.12	0.050	0.85	0.84
189-2-TL	14,087	160.69	0.046	0.77	0.76
192-1-TL	15,538	126.49	0.044	0.45	0.47
192-2-TL	14,421	139.78	0.048	0.61	0.62
192-3-TL	13,220	114.81	0.050	0.47	0.44
Mean	13,370	153.39	0.049	0.81	0.77
SD	1207	20.76	0.003	0.23	0.19
CoV (%)	9	14	7	29	24

**Table 5:** Logistic function parameters (*A*_1_, *A*_2_, *p*, and *w*_I0_), maximum stress (*σ*_Iu_) and relative displacement (*w*_Iu_), as determined by CBBM equations, from specimens with the RL crack system.

Ref	*A*_1_ (N/mm)	*A*_2_ (N/mm)	*p* (-)	*w*_I0_ (mm)	*G*_law,I_ (N/mm)	*σ*_Iu_ (MPa)	*w*_Iu_ (mm)
140-1-RL	0.044	1.04	2.93	0.030	1.00	26.94	0.024
144-1-RL	0.039	0.82	2.80	0.019	0.78	32.21	0.015
144-2-RL	0.024	0.49	2.30	0.023	0.46	14.21	0.015
161-1-RL	0.029	0.93	2.20	0.023	0.91	27.19	0.014
161-3-RL	0.016	0.78	2.02	0.020	0.75	25.39	0.011
176-1-RL	0.026	0.67	2.46	0.018	0.64	26.35	0.013
176-2-RL	0.010	0.78	1.42	0.026	0.76	18.00	0.008
176-3-RL	0.025	1.10	1.58	0.074	1.05	8.86	0.029
189-1-RL	0.019	0.73	2.29	0.051	0.70	9.79	0.034
189-2-RL	0.028	0.97	1.72	0.044	0.92	13.11	0.020
192-1-RL	0.024	0.63	2.37	0.016	0.61	27.41	0.011
192-2-RL	0.043	0.66	3.60	0.019	0.62	32.11	0.016
192-3-RL	0.035	0.58	2.69	0.012	0.55	34.78	0.009
Mean	0.028	0.78	2.34	0.029	0.75	22.80	0.017
SD	0.010	0.18	0.59	0.018	0.18	8.90	0.008
CoV (%)	37	23	25	61	24	39	47

**Table 6:** Logistic function parameters (*A*_1_, *A*_2_, *p*, and *w*_I0_), maximum stress (*σ*_Iu_) and relative displacement (*w*_Iu_), as determined by CBBM equations, from specimens with a TL crack system.

Ref	*A*_1_ (N/mm)	*A*_2_ (N/mm)	*p* (-)	*w*_I0_ (mm)	*G*_law,I_ (N/mm)	*σ*_I,u_ (MPa)	*w*_Iu_ (mm)
140-1-TL	0.027	0.96	1.93	0.038	0.93	15.65	0.021
140-2-TL	0.012	0.94	1.83	0.056	0.92	10.31	0.029
144-1-TL	0.016	0.80	2.25	0.034	0.78	15.86	0.022
161-1-TL	0.023	1.16	1.92	0.056	1.13	12.92	0.031
176-1-TL	0.013	0.64	1.74	0.031	0.61	12.33	0.015
176-2-TL	0.007	0.73	1.73	0.043	0.70	10.31	0.020
176-3-TL	0.025	0.80	1.97	0.074	0.76	6.77	0.042
189-1-TL	0.015	0.87	1.70	0.063	0.85	8.35	0.029
189-2-TL	0.028	0.79	2.07	0.061	0.76	8.28	0.037
192-1-TL	0.023	0.45	2.26	0.036	0.43	8.35	0.024
192-2-TL	0.011	0.61	1.98	0.080	0.58	4.83	0.045
192-3-TL	0.007	0.42	2.26	0.042	0.41	6.78	0.027
Mean	0.017	0.76	1.97	0.051	0.74	10.06	0.028
SD	0.008	0.21	0.21	0.016	0.21	3.52	0.009
CoV (%)	44	28	10	32	28	35	32
